# Obacunone Retards Renal Cyst Development in Autosomal Dominant Polycystic Kidney Disease by Activating NRF2

**DOI:** 10.3390/antiox11010038

**Published:** 2021-12-24

**Authors:** Zhiwei Qiu, Jinzhao He, Guangying Shao, Jiaqi Hu, Xiaowei Li, Hong Zhou, Min Li, Baoxue Yang

**Affiliations:** State Key Laboratory of Natural and Biomimetic Drugs, Department of Pharmacology, School of Basic Medical Sciences, Peking University, Beijing 100191, China; 1410305218@pku.edu.cn (Z.Q.); 18200288030@163.com (J.H.); 18210466583@163.com (G.S.); 1810301131@pku.edu.cn (J.H.); xiaowei@bjmu.edu.cn (X.L.); zhouhong@bjmu.edu.cn (H.Z.); leemin@bjmu.edu.cn (M.L.)

**Keywords:** obacunone, autosomal dominant polycystic kidney disease, NRF2, glutathione peroxidase, lipid peroxidation, MAPK, mTOR

## Abstract

Autosomal dominant polycystic kidney disease (ADPKD) is a common inherited disease characterized by progressive enlargement of fluid-filled cysts derived from renal tubular epithelial cells, which has become the fourth leading cause of end-stage renal diseases. Currently, treatment options for ADPKD remain limited. The purpose of this study was to discover an effective therapeutic drug for ADPKD. With virtual screening, Madin-Darby canine kidney (MDCK) cyst model, embryonic kidney cyst model and kidney-specific *Pkd1* knockout mouse (PKD) model, we identified obacunone as a candidate compound for ADPKD drug discovery from a natural antioxidant compound library. *In vitro* experiments showed that obacunone significantly inhibited cyst formation and expansion of MDCK cysts and embryonic kidney cysts in a dose-dependent manner. *In vivo*, obacunone treatment significantly reduced the renal cyst development in PKD mice. Western blot and morphological analysis revealed that obacunone served as a NRF2 activator in ADPKD, which suppressed lipid peroxidation by up-regulating GPX4 and finally restrained excessive cell proliferation by down-regulating mTOR and MAPK signaling pathways. Experimental data demonstrated obacunone as an effective renal cyst inhibitor for ADPKD, indicating that obacunone might be developed into a therapeutic drug for ADPKD treatment.

## 1. Introduction

Autosomal dominant polycystic kidney disease (ADPKD) is a human inherited disease with an estimated prevalence of between one in 2500 to one in 1000 individuals [1]. It is characterized by progressive enlargement of fluid-filled cysts derived from renal tubular epithelial cells. Massive cysts gradually compress renal parenchyma, destroy the normal renal structures, and eventually cause the loss of kidney function [2]. ADPKD is mostly caused by mutations in one of two genes, *Pkd1* or *Pkd2*, which encode the proteins polycystin-1 (PC1) or polycystin-2 (PC2), respectively [3]. The dysfunction of PC1 or PC2 leads to an abnormal decrease in intracellular Ca^2+^ concentration, which triggers cystogenesis by promoting excessive epithelial cell proliferation and fluid secretion [1].

With the progress of ADPKD, it eventually evolves into end-stage renal disease (ESRD), which seriously endangers the lives of patients [4,5]. Unfortunately, the therapies for ADPKD are still limited. Tolvaptan is the only drug approved by Food and Drug Administration (FDA) for ADPKD treatment at present, but the hepatotoxicity and side effects limit its application [6]. Prophylactic and symptomatic treatments remain the effective principles to retard renal cyst development. Patients with ESRD have to undergo replacement therapy [7]. These facts have brought a huge economic burden to patients and society [8]. Therefore, it is of great significance to develop drugs with a potent therapeutical effect, few side effects and a reasonable price for ADPKD treatment.

The advantages of low toxicity and easy availability make natural compounds ideal for ADPKD therapeutic drug development. In previous studies, we had identified several natural compounds that showed effective inhibitory effects on cyst development in ADPKD experimental models, such as curcumin [9], ginkgolide B [10], Ganoderma triterpenes [11] and cardamonin [12]. The druggabilities of these compounds are still undergoing evaluation. Nevertheless, it is an effective way to screen druggable compounds based on targeting to signaling pathways that are important for the progression of ADPKD. Oxidative stress is a common pathological mechanism in the ADPKD [13]. Elevated levels of oxidative stress in renal tissues were found in both fast and slow progressing ADPKD animal models and patients [13,14,15]. These facts make it feasible to hunt for ADPKD therapeutic drugs from natural antioxidant compounds.

In this study, we identified obacunone from a library of natural antioxidant compounds, which showed cyst inhibitory effect in Madin-Darby canine kidney (MDCK) cyst model, embryonic kidney cyst model and kidney-specific *Pkd1* knockout mouse (PKD) model. As a potent antioxidant, obacunone activated NRF2 in the early stage of ADPKD, which suppressed lipid peroxidation and finally retarded excessive cell proliferation by down-regulating mTOR and MAPK signaling pathways. Our study suggests that obacunone might be developed into a therapeutic drug for ADPKD.

## 2. Materials and Methods

### 2.1. Cell Culture

Type I Madin-Darby canine kidney (MDCK) cells (ATCC Number: CRL-2936) were cultured in a 5% CO_2_ and 37 °C humidified incubator with Dulbecco’s modified Eagle’s medium (DMEM) (Invitrogen, Carlsbad, CA, USA, Cat# 12800082) containing 10% fetal bovine serum (FBS) (Gibco, Carlsbad, CA, USA, Cat# 10099141C), 100 μg/mL streptomycin and 100 U/mL penicillin (Gibco, Carlsbad, CA, USA, Cat# 15140148). Mouse inner medullary collecting duct cells (mIMCD-3) (ATCC Number: CRL-2123) were cultured in a 5% CO_2_ and 37 °C humidified incubator with DMEM/F-12 (Invitrogen, Carlsbad, CA, USA, Cat# 12400024) containing 10% FBS, 100 μg/mL streptomycin and 100 U/mL penicillin.

### 2.2. Cell Counting Kit-8 Assay

MDCK cells were counted and seeded in quintuplicate on a 96-well plate at a density of 5000 cells/well. The cells were cultured in a 5% CO_2_ and 37 °C humidified incubator with DMEM (containing 10% FBS) for 24 h. Then, cells in different groups were exposed to candidate compounds for 24 h, respectively. After treatment, 100 μL 5% cell counting Kit-8 (CCK-8) solution (Dojindo, Kumamoto, Japan, Cat# CK04) was added to each well and incubated for additional 1.5 h. Finally, the absorbance at the wavelength of 450 nm was measured by a microplate reader (Biotek, Santa Clara, CA, USA, MQX200). Blank wells (only containing DMEM and CCK-8) and control wells (containing untreated cells, DMEM and CCK-8) were also detected.

### 2.3. MDCK Cyst Model

MDCK cyst model was performed as previously described [11]. Briefly, MDCK cells were cultured in Type I collagen (Sigma-Aldrich, Saint Louis, MO, USA, Cat# C2124) plated in 24-well plates at a density of 800 cells/well. DMEM/F-12 medium containing 10 μM forskolin (Sigma-Aldrich, Saint Louis, MO, USA, Cat# F6886), without or with candidate compounds, was added to each well and replaced every 12 h.

To detect the influence of obacunone (Selleck, Houston, TX, USA, Cat# S3784) on the formation of MDCK cysts, different concentrations of obacunone were supplied into the medium at day 0, and the numbers of cell colonies and cysts (larger than 50 μm) in different groups were counted using a Nikon microscope (Nikon, Tokyo, Japan, ECLIPSE Ti2-U) at day 6. The cyst formation rate was calculated by dividing the number of cysts by the number of total colonies, so as to reflect the effect of obacunone on cyst formation. Three repeating wells were set for each dose.

To detect the influence of candidate compounds on the growth of MDCK cysts, different compounds in candidate concentration were supplied into the medium at day 4 (before micrography). And micrographs of the cysts (30/group) were taken at day 4, 6, 8, 10 and 12. Diameters of the cysts were measured using ImageJ software (National Institutes of Health, Bethesda, MD, USA).

### 2.4. Embryonic Kidney Cyst Model

Embryonic kidney cyst model was performed as described previously [11]. Briefly, kidneys from wild-type C57BL/6 mice at embryonic day 13.5 were isolated and placed on Transwells (Corning, Corning, NY, USA, Cat# 3493). The embryonic kidneys were incubated with DMEM/F12 containing 2 mM L-glutamine (Aladdin, Shanghai, China, Cat# G105425), 10 mM HEPES, 5 μg/mL insulin (Sigma-Aldrich, Saint Louis, MO, USA, Cat# I2643), 5 μg/mL transferrin (Sigma-Aldrich, Saint Louis, MO, USA, Cat# T8158), 2.8 nM sodium selenate decahydrate (Santa Cruz, Dallas, TX, USA, Cat# sc-236924), 25 ng/mL prostaglandin E1 (Sigma-Aldrich, Saint Louis, MO, USA, Cat# P5515), 32 pg/mL T3 (Sigma-Aldrich, Saint Louis, MO, USA, Cat# T2877), 250 U/mL penicillin, 250 μg/mL streptomycin and 100 μM 8-Br-cAMP (Sigma-Aldrich, Saint Louis, MO, USA, Cat# B5386) (the blank group did not contain 8-Br-cAMP). Different compounds in candidate concentration were supplied to the medium at day 0, and medium was replaced every 12 h. Kidneys were photographed using a Nikon microscope at day 0, 2, 4 and 6. Cyst index was calculated by comparing the total cyst area to total kidney area using the ImageJ software (National Institutes of Health, Bethesda, MD, USA).

### 2.5. Animals

*Pkd1^flox/flox^* mice (from the Yale PKD Center) and *Ksp-Cre* transgenic mice (from the University of Texas Southwestern O’Brien Center) on C57BL/6 background were generated as described previously [16]. Kidney-specific *Pkd1* knockout mice (with *Pkd1^flox/flox^*; *Ksp-Cre* genotype) were generated by cross-breeding *Pkd1^flox/flox^* mice with *Ksp-Cre* mice. Neonatal mice (1 day old) were genotyped by genomic PCR. In order to explore the therapeutical effect of obacunone on ADPKD, 100 mg/kg obacunone (dissolved in saline with 1/1000 DMSO) was injected intraperitoneally every day from postnatal day 1 to day 4. At postnatal day 5, kidneys were removed, weighed and fixed for histologic examination. In order to explore the activation effect of obacunone on NRF2 in the early stage of ADPKD, 100 mg/kg obacunone was injected intraperitoneally every day from postnatal day 1 to day 2. At postnatal day 3, kidneys were removed, weighed and fixed for histologic examination.

### 2.6. Histology

Kidneys were collected and processed as described previously [12]. Briefly, kidneys were fixed by 4% paraformaldehyde (Sigma-Aldrich, Saint Louis, MO, USA, Cat# 158127), gradiently dehydrated by alcohol (Tong Guang, Beijing, China, Cat# 104022) and embedded in paraffin (Leica, Wetzlar, Germany, Cat# 39601095). 5 μm sections were cut and stained with hematoxylin (Amresco, Radnor, PA, USA, Cat# 0701) and eosin (Amresco, Radnor, PA, USA, Cat# 0109). Kidney sections were photographed using a Nikon microscope. Cyst index was calculated by comparing the total cyst area to total kidney area using the ImageJ software (National Institutes of Health, Bethesda, MD, USA).

### 2.7. Immunofluorescence Staining

For kidney tissue, kidneys were fixed by 4% paraformaldehyde, gradiently dehydrated by sucrose (Aladdin, Shanghai, China, Cat# S112228) and embedded in optimal cutting temperature compound (SAKURA, Tokyo, Japan, Cat# 4583). 5 μm sections were cut. 5% (*w*/*v*) bovine serum albumin was used to block the sections at room temperature for 1 h. Then, the sections were incubated with primary antibodies against Ki-67 (Abcam, Cambridge, UK, Cat# ab15580, 1:500 dilution) at 4 °C overnight. The next day, sections were washed 3 times and incubated with Cy3-conjugated Affinipure goat anti-rabbit IgG(H+L) (Proteintech, Wuhan, China, Cat# SA00009-2, 1:100 dilution) secondary antibodies or *Lotus tetragonolobus lectin* (LTL, Vector Laboratories, Burlingame, CA, USA, Cat# FL-1321, 1:400 dilution) and *Dolichos biflorus agglutinin* (DBA) (Vector Laboratories, Burlingame, CA, USA, Cat#, RL-1032, 1:400 dilution) for 1 h. DAPI (Sigma, Saint Louis, MO, USA, Cat# D9542) was used to stain nuclei. Images were captured with a Nikon fluorescence microscope (Nikon, Tokyo, Japan, ECLIPSE Ti2-U). The unit areas of Ki-67 positive cells were calculated using ImageJ software (National Institutes of Health, Bethesda, MD, USA).

For mIMCD-3 cells, cells were seeded into glass bottom dish (Cellvis, Mountain View, CA, USA, Cat# D35-10-1-N). After stimulation without or with forskolin or/and obacunone, 4% paraformaldehyde was added to fix the cells for 15 min. 5% (*w*/*v*) bovine serum albumin was used to block the sections at room temperature for 1 h. Then, the cells were incubated with primary antibodies against NRF2 (ABclonal, Wuhan, China, Cat# A0674, 1:200 dilution) and β-actin (ABclonal, Wuhan, China, Cat# AC004, 1:200 dilution) at 4 °C overnight. The next day, cells were washed 3 times and incubated with fluorescein (FITC)-conjugated affinipure goat anti-mouse IgG(H+L) (Proteintech, Wuhan, China, Cat# SA00003-1, 1:100 dilution) and Cy3-conjugated affinipure goat anti-rabbit IgG(H+L) (Proteintech, Wuhan, China, Cat# SA00009-2, 1:100 dilution) secondary antibodies. DAPI (Sigma, Saint Louis, MO, USA, Cat# D9542) was used to label nuclei. Images were captured by a Nikon confocal microscope (Nikon, Tokyo, Japan, TiE-A1 plus).

### 2.8. Western Blot Analysis

Total kidney protein was extracted using RIPA lysis buffer (Applygen, Beijing, China, Cat# C1053) containing 4% protease inhibitor cocktail (Roche, South San Francisco, CA, USA, Cat# 11873580001) and 1% protein phosphatase inhibitor (Applygen, Beijing, China, Cat# P1260). Nucleoprotein was extracted using a nucleoprotein extraction kit (Bestbio, Shanghai, China, Cat# BB-3102).

Protein samples were separated based on molecular weight through SDS-polyacrylamide gel electrophoresis and transferred to polyvinylidene difluoride membranes (PALL, Beijing, China, Cat# BSP0161). The membranes were blocked with blocking buffer (TBS, 0.1% Tween-20, 5% non-fat milk or 2% BSA) for 2 h at room temperature and incubated with primary antibodies against NRF2 (ABclonal, Wuhan, China, Cat# A0674, 1:1000 dilution), Lamin B1 (ABclonal, Wuhan, China, Cat# A1910, 1:1000 dilution), 4-HNE (Bioss, Beijing, China, Cat# bs-6313R, 1:1000 dilution), GPX4 (ABclonal, Wuhan, China, Cat# A13309, 1:1000 dilution), H-Ras (ABclonal, Wuhan, China, Cat# A19619, 1:1000 dilution), B-Raf (ABclonal, Wuhan, China, Cat# A15033, 1:1000 dilution), p-ERK (ABclonal, Wuhan, China, Cat# AP0974, 1:1000 dilution), ERK (ABclonal, Wuhan, China, Cat# A16686, 1:1000 dilution), p-mTOR (Cell Signaling Technology, Danvers, MA, USA, Cat# 5536S, 1:1000 dilution), mTOR (Cell Signaling Technology, Danvers, MA, USA, Cat# 2983, 1:1000 dilution), p-S6 (ABclonal, Wuhan, China, Cat# AP0537, 1:1000 dilution), S6 (ABclonal, Wuhan, China, Cat# A6058, 1:1000 dilution), PCNA (Cell Signaling Technology, Danvers, MA, Cat# 2586, 1:1000 dilution), β-actin (ABclonal, Wuhan, China, Cat# AC004, 1:10,000 dilution) at 4 °C, respectively. Afterwards, HRP-conjugated goat anti-rabbit IgG (H+L) (Immunoway, Suzhou, China, Cat# RS0002) or HRP-conjugated goat anti-mouse IgG (Easybio, Beijing, China, Cat# BE0102) were incubated for 60 min at room temperature. The ECL kit (Meilunbio, Dalian, China, Cat# MA0186) was used to detect protein expression signal intensity through a chemiluminescence detection system (Syngene, Frederick, MD, USA, GeneGnome XRQ). Relative protein expression levels were quantified by the ImageJ software (National Institutes of Health, Bethesda, MD, USA).

### 2.9. GSH-Px, GSH, and MDA Assay

The activity of GSH-Px and content of GSH and MDA in the kidney were detected using commercial kits as follows: glutathione peroxidase (GSH-PX) assay kit (Nanjing Jiancheng, Nanjing, China, Cat# A005-1-2), reduced glutathione (GSH) assay kit (Nanjing Jiancheng, Nanjing, China, Cat# A006-2-1), malondialdehyde (MDA) assay kit (Nanjing Jiancheng, Nanjing, China, Cat# A003-1-2). Detection procedure is carried out in strict accordance with the protocols provided by the kits.

### 2.10. Statistical Analyses

All results were represented as Mean ± SEM. Statistical analyses were performed with GraphPad Prism 8.0 software. Data were first analyzed with the D’Agostino–Pearson omnibus normality test to verify their distribution. Parametric tests were performed where data obeyed gaussian distribution. Data involving in two groups were analyzed using two-tailed student’s *t* test. When more than two experimental groups were compared, the data were analyzed using one-way ANOVA with Tukey multiple comparison tests. Data that did not obey gaussian distribution were analyzed using the Kruskal–Wallis test. *p*-value < 0.05 was considered to be statistically significant.

## 3. Results

### 3.1. Obacunone Is Identified as an Inhibitor of Renal Cysts

**Figure 1:**
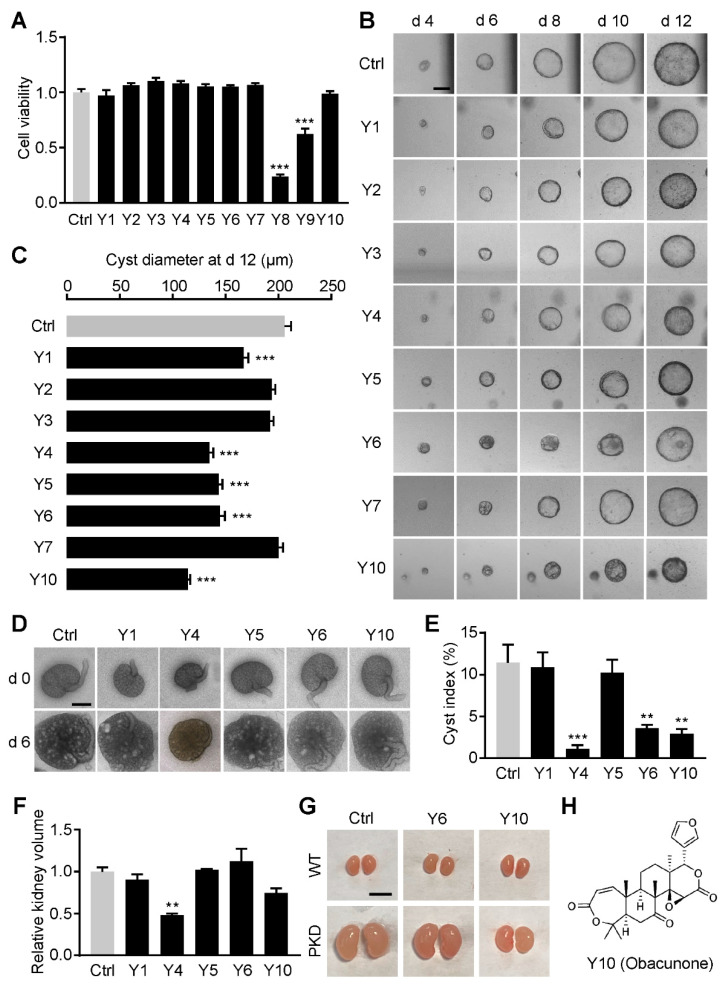
Identification of obacunone as a potent renal cyst inhibitor. (**A**) Cytotoxicity of 12.5 μM candidate compounds on MDCK cells. n = 5. (**B**) Representative images of MDCK cysts treated without (Ctrl) or with 12.5 μM candidate compounds. Bar = 100 μm. (**C**) Diameters of MDCK cysts at day 12. n = 30. (**D**) Representative images of embryonic kidneys. Bar = 500 μm. (**E**) Cyst indices of embryonic kidneys at day 6. n = 6. (**F**) Volumes of embryonic kidneys at day 6. n = 6. (**G**) Representative images of kidneys from wild-type (WT) or kidney specific *Pkd1* knockout mice (PKD) treated without or with 100 mg/(kg·d) candidate compounds. Bar = 0.5 cm. (**H**) Chemical structure of Y10. Data are presented as means ± SEM. ** *p* < 0.01, *** *p* < 0.001, versus Ctrl.

By a virtual screening based on the effect on oxidative stress response-related signaling pathways, such as KEAP1/NRF2, NOX4 and PERK, 10 candidate compounds (Y1–Y10) were selected from a natural compound library at Selleck. CCK-8 assay showed that 12.5 μM Y8 and Y9 significantly reduced MDCK cell viability (Figure 1A), which were excluded in the further studies due to their cytotoxicity.

The remaining eight compounds (12.5 μM) were investigated in a MDCK cyst model to detect their cyst inhibitory effect. MDCK cells form cysts in type I collagen with 10 μM forskolin stimulation, which is widely used as an *in vitro* model of ADPKD. As shown in Figure 1B,C, 12.5 μM Y1, Y4, Y5, Y6 or Y10 significantly inhibited the expansion of MDCK cysts. These five compounds (12.5 μM) were further detected with an embryonic kidney cyst model to evaluate the cyst inhibitory activity at the organ level. E13.5 embryonic kidneys cultured in transwell form cysts with 100 μM 8-Br-cAMP stimulation, which is used as an *ex vivo* model of ADPKD. As shown in Figure 1D,E, Y4, Y6 or Y10 stimulation significantly retarded cyst development in embryonic kidney. However, Y4 inhibited the growth of embryonic kidneys (Figure 1D,F), indicating its toxic effect on kidney development.

*In vivo* inhibiting effects of Y6 and Y10 on renal cyst development were determined in a kidney-specific *Pkd1* knockout mouse (*Pkd1^flox/flox^*; *Ksp-Cre*, PKD) model. PKD mice develop acutely progressive ADPKD, in which renal cysts appear at postnatal day 1 and progressively enlarge, which causes death of the animal around postnatal day 14 [12]. As shown in Figure 1G, PKD kidneys were much larger than wild-type ones. Y10 (100 mg/(kg·d)) reduced the kidney volume of PKD mice without affecting kidney development in wild-type mice. These results indicate that Y10 has potent cyst inhibition activity and little cytotoxicity. The chemical structure of Y10, which is called obacunone, is shown in Figure 1H.

### 3.2. Obacunone Dose-Dependently Inhibits MDCK Cyst Formation and Enlargement

**Figure 2:**
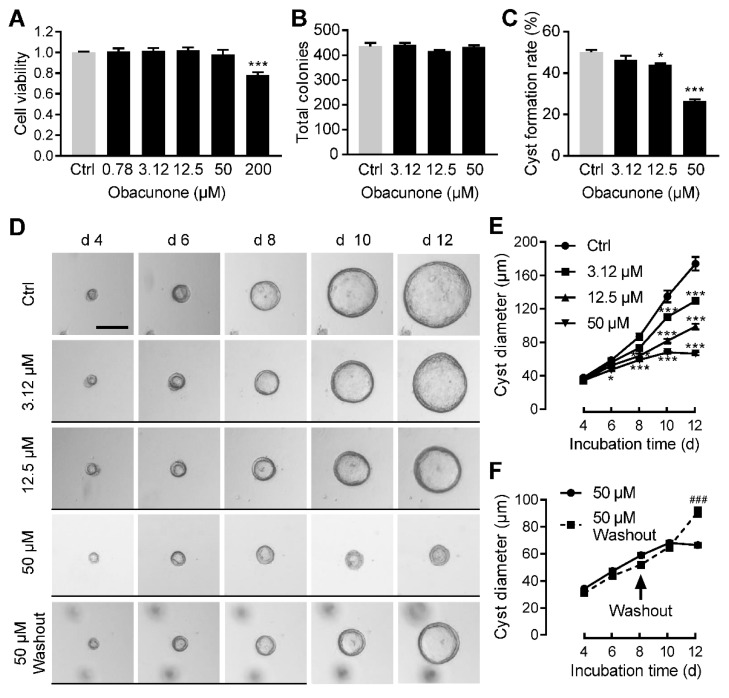
Inhibitory effect of obacunone on MDCK cyst formation and expansion. (**A**) Viability of MDCK cells treated without (Ctrl) or with obacunone. n = 5. (**B**) Number of total colonies in MDCK cyst model. n = 3. (**C**) Cyst formation rate. n = 3. (**D**) Representative images of MDCK cysts. Narrow black lines indicate obacunone treatment. Bar = 100 μm. (**E**) Diameters of MDCK cysts showing dose response of obacunone. n = 30. (**F**) Diameter of MDCK cysts showing reversible effect of obacunone. Arrow indicates the time of washout obacunone. n = 30. Data are presented as means ± SEM. * *p* < 0.05, *** *p* < 0.001 versus Ctrl. ^###^ *p* < 0.001 versus 50 μM obacunone treatment without washout.

CCK-8 assay showed that obacunone had no significant cytotoxicity to MDCK cells at concentration of 50 μM and below (Figure 2A). We first evaluated the effect of obacunone on MDCK cyst formation. MDCK cells were incubated in type I collagen without or with obacunone at 3.12, 12.5 or 50 μM from day 0 to day 6. At day 6, the numbers of total colonies (including both cell colonies and cysts) were similar among all groups (Figure 2B). However, numbers of cysts (diameter larger than 50 μm) were reduced by obacunone treatment in a dose-dependent manner with inhibitory rates of ~7.3%, ~12.2% and ~46.9%, respectively (Figure 2C), indicating that obacunone significantly inhibited the formation of MDCK cysts.

To evaluate the effect of obacunone on the cyst enlargement, MDCK cysts were treated without or with obacunone from day 5 to day 12. It was found that cyst growth was inhibited by obacunone in a dose-dependent manner (Figure 2D,E). Interestingly, when obacunone was washed out at day 8, inhibited MDCK cysts regrew again (Figure 2F), suggesting that the inhibitory effect of obacunone on MDCK cyst growth was reversible.

### 3.3. Obacunone Inhibits Cyst Development in Embryonic Kidney

**Figure 3:**
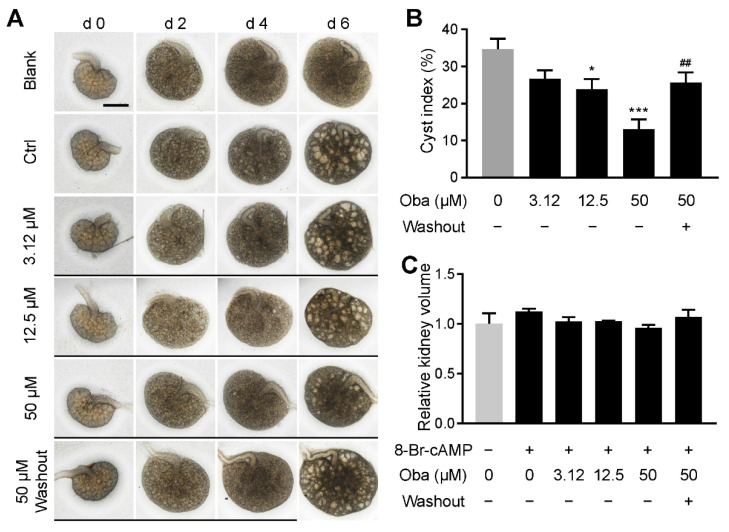
Inhibitory effect of obacunone on the enlargement of embryonic kidney cyst. (**A**) Representative images of embryonic kidneys treated without (Ctrl) or with obacunone. Narrow black lines indicate obacunone treatment. Bar = 500 μm. (**B**) Cyst indices (cyst area to embryonic kidney area). (**C**) Relative kidney volumes of embryonic kidneys on day 6. Data are presented as means ± SEM. n = 6. * *p* < 0.05, *** *p* < 0.001 versus Ctrl. ^##^ *p* < 0.01 versus 50 μM obacunone treatment.

To confirm the inhibitory effect of obacunone at the organ level, we detected cyst development in the embryonic kidney cyst model. Experimental results showed that obacunone dose-dependently inhibited the renal cysts enlargement, which is consistent with the observation in the MDCK cyst model. Similarly, the inhibitory effect of 50 μM obacunone on the cyst growth was abolished when obacunone was washed out at day 4 (Figure 3A,B), confirming the reversible therapeutical effect of obacunone on ADPKD. However, obacunone had no adverse effect on the development of embryonic kidneys, as the obacunone-treated embryonic kidneys had similar volumes as the ‘blank’ (i.e., no 8-Br-cAMP and no obacunone) and ‘control’ (i.e., no obacunone) groups (Figure 3A,C).

### 3.4. Obacunone Retards Renal Cyst Development in PKD Mice

**Figure 4:**
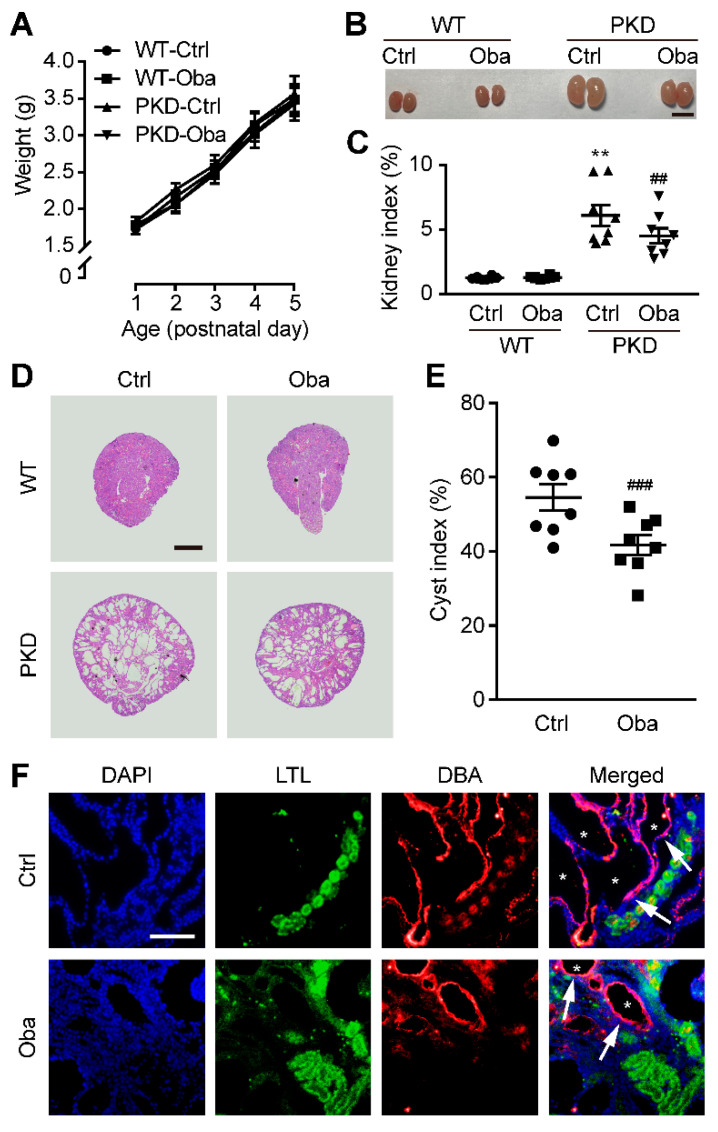
Inhibitory effect of obacunone on cyst development in PKD mice. (**A**) Weight curves of mice. (**B**) Representative images of the kidneys. Bar = 1 cm. (**C**) Kidney indices. (**D**) Hematoxylin- and eosin-stained pictures of kidneys. Bar = 400 μm. (**E**) Cyst indices. (**F**) LTL (green), DBA (red) and DAPI (blue) fluorescence staining of PKD kidneys. Arrows indicate the collecting ducts, asterisks represent the cyst area. Bar = 50 μm. Data are presented as means ± SEM. n = 8. ** *p* < 0.01 versus WT-Ctrl. ^##^ *p* < 0.01, ^###^ *p* < 0.001 versus PKD-Ctrl.

We further investigated the cyst inhibitory effect of obacunone *in vivo*. Treatment with obacunone (100 mg/kg, injected intraperitoneally every day from postnatal day 1 to day 4) did not affect the size, growth and behavior of mice (Figure 4A), but significantly retarded the enlargement of bilateral kidneys of PKD mice (Figure 4B,C). Kidney sections showed that obacunone significantly reduced the kidney cyst indices of PKD mice (Figure 4D,E). Moreover, labeling collecting ducts by DBA and proximal tubules by LTL with immunostaining, we found that the renal cysts were mainly formed in collecting ducts rather than proximal tubules (Figure 4F).

### 3.5. Obacunone Activates NRF2 in the Early Stage of ADPKD

**Figure 5:**
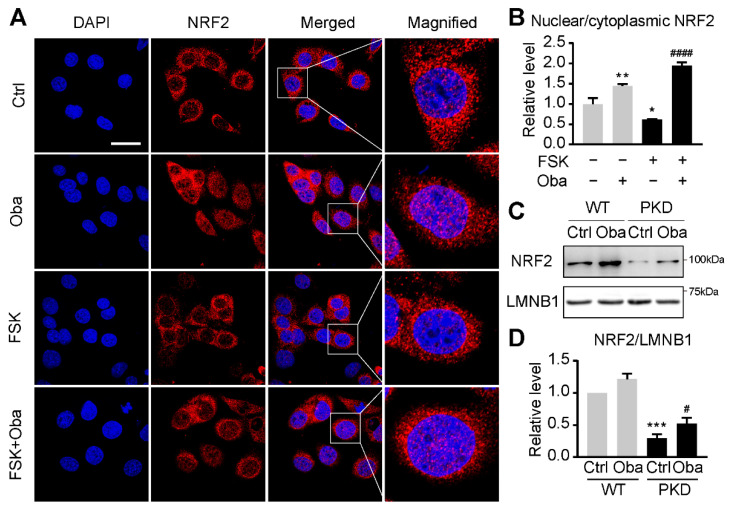
Effect of obacunone on NRF2 activity. (**A**) Immunofluorescence of NRF2 (red) and DAPI (blue) staining in mIMCD-3 cells treated without (Ctrl) or with forskolin (FSK) or obacunone (Oba). Bar = 10 μm. (**B**) Quantification of fluorescence intensities of NRF2 as in (**A**). n = 12 cells. (**C**) Representative Western blots of nuclear NRF2 in the kidneys of wild-type (WT) and PKD mice (PKD) treated without or with obacunone. (**D**) Quantification of nuclear NRF2 expression levels as in (**C**). Data are presented as means ± SEM. n = 6. * *p* < 0.05, ** *p* < 0.01 versus Ctrl. ^####^ *p* < 0.0001 versus FSK. *** *p* < 0.001 versus WT-Ctrl. ^#^ *p* < 0.05 versus PKD-Ctrl.

Previous studies have reported that obacunone is an effective activator of NRF2 [17,18,19,20] that plays an important role in ROS scavenging [21,22,23]. NRF2 knockout promotes the development of renal cysts in ADPKD mouse models [24]. Therefore, we investigated the effect of obacunone on NRF2 activation. Out of our expectation, although obacunone promoted nuclear translocation of NRF2 in FSK-stimulated mIMCD-3 cells (Figure 5A,B), obacunone treatment for four days did not up-regulate intranuclear NRF2 in PKD kidneys (data not shown). Considering that oxidative stress response usually occurs before cyst formation in ADPKD [15], and the activity of NRF2 often changes dynamically during the disease progression [25], we speculated that obacunone might activate NRF2 in the early stage of ADPKD.

To verify our hypothesis, we explored the activation effect of obacunone on NRF2 in the early stage of ADPKD *in vivo*. PKD mice were treated with obacunone (100 mg/kg, injected intraperitoneally every day) from postnatal day 1 to day 2. The kidneys were sampled at postnatal day 3 and the expression level of intranuclear NRF2 was detected immediately. The experimental results showed that short-term obacunone treatment significantly up-regulated the expression of NRF2 in the renal cell nucleus of PKD mice (Figure 5C,D), which indicates that obacunone activates NRF2 in the early stage of ADPKD.

### 3.6. Obacunone Suppresses Lipid Peroxidation in ADPKD

**Figure 6:**
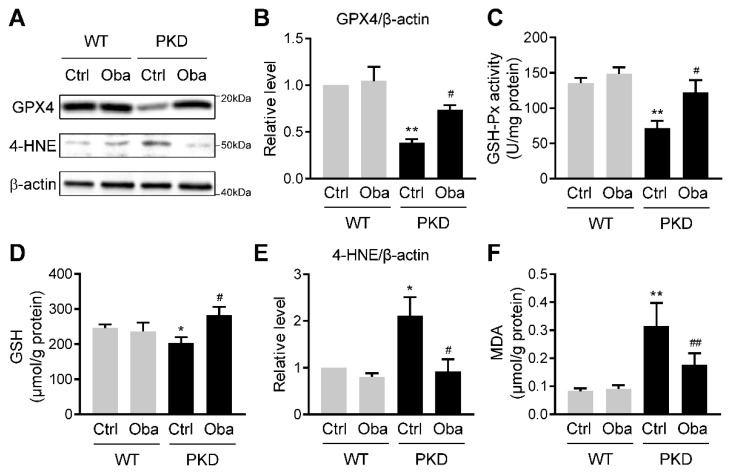
Inhibition effect of obacunone on lipid peroxidation. (**A**) Representative Western blots of GPX4 and 4-HNE in the kidneys of wild-type (WT) and PKD (PKD) mice treated without (Ctrl) or with obacunone (Oba). (**B**) Quantification of GPX4 expression levels. (**C**) GSH-Px activity. (**D**) GSH content. (**E**) Quantification of 4-HNE expression levels. (**F**) MDA content. Data are presented as means ± SEM. n = 6. * *p* < 0.05, ** *p* < 0.01 versus WT-Ctrl. ^#^ *p* < 0.05, ^##^ *p* < 0.01 versus PKD-Ctrl.

To further explore the effect of obacunone-activated NRF2 on the development of ADPKD, we detected the expression of the NRF2 target gene *glutathione peroxidase 4* (*GPX4*) in PKD mice at postnatal day 5. Western blot results showed that obacunone significantly up-regulated the GPX4 expression in PKD kidneys (Figure 6A,B), which was accompanied by an enhancement of glutathione peroxidase (GSH-Px) activity (Figure 6C). The decrease of glutathione (the substrate of GPX4) in ADPKD was also reversed by obacunone treatment (Figure 6D).

Previous studies have confirmed that GPX4 is a key factor in regulating lipid peroxidation [26], which plays a catalytic role in promoting the ADPKD progression [14]. Hence, we investigated the effect of obacunone on lipid peroxidation in PKD mice. As expected, the levels of 4-hydroxy-2-nonenal (4-HNE)-modified protein (reflecting the content of 4-HNE) (Figure 6A,E) and content of malondialdehyde (MDA) (Figure 6F) were increased in kidneys from control PKD mice and reduced with obacunone treatment.

### 3.7. Obacunone Inhibits Abnormal Cell Proliferation by Down-Regulating mTOR and MAPK Signaling Pathways

**Figure 7:**
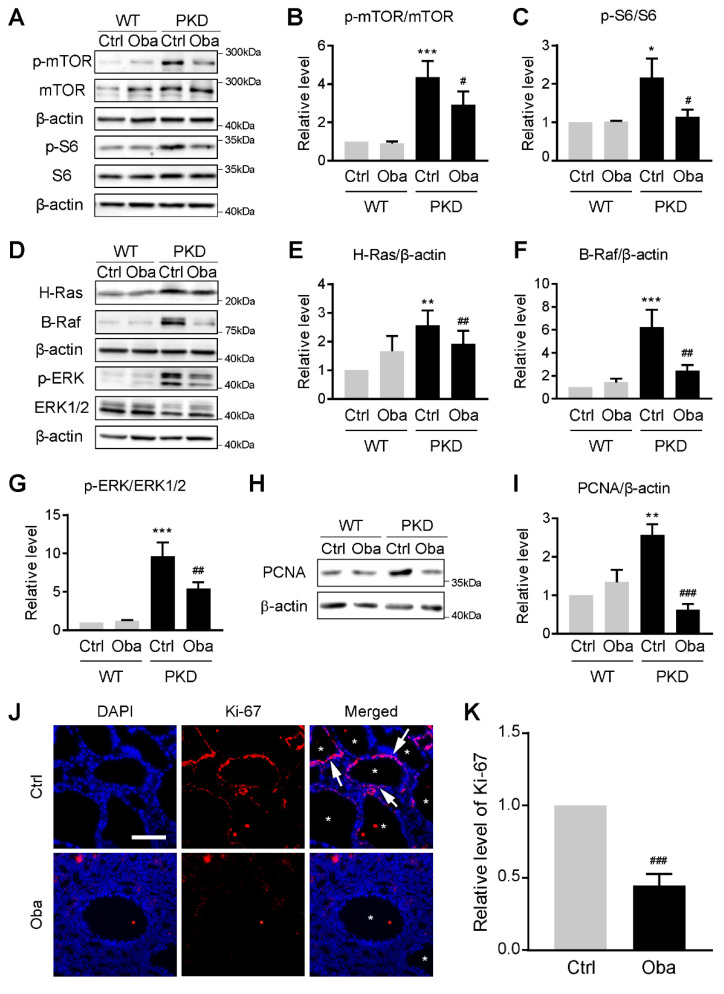
Inhibitory effect of obacunone on abnormal cell proliferation. (**A**) Representative Western blots of mTOR signaling pathway proteins in the kidneys of wild-type (WT) and PKD mice (PKD) treated without (Ctrl) or with obacunone (Oba). (**B**) Relative phosphorylation levels of mTOR protein. (**C**) Relative phosphorylation levels of S6 protein. (**D**) Representative Western blots of MAPK signaling pathway proteins. (**E**) Quantification of H-Ras protein expression levels. (**F**) Quantification of B-Raf protein expression levels. (**G**) Relative phosphorylation levels of ERK protein. (**H**) Representative Western blots of PCNA. (**I**) Quantification of PCNA protein expression levels. (**J**) Immunofluorescence staining of Ki-67 (red) and DAPI (blue) in the PKD kidneys. Arrows represent the Ki-67 positive cells, asterisks represent the cyst area. Bar = 50 μm. (**K**) Quantification of Ki-67 positive cells. Data are presented as means ± SEM. n = 6. * *p* < 0.05, ** *p* < 0.01, *** *p* < 0.001 versus WT-Ctrl. ^#^ *p* < 0.05, ^##^ *p* < 0.01, ^###^ *p* < 0.001 versus PKD-Ctrl.

Large amounts of lipid peroxides (LPOs), such as MDA and 4-HNE [27], have been reported as the activators of mTOR [28,29] and MAPK [30,31,32] signaling pathways, which are involved in the ADPKD progression. Therefore, we investigated the effect of obacunone on these two signaling pathways. It was found that obacunone significantly inhibited the expression of H-Ras, B-Raf and phosphorylation of ERK, mTOR and S6 in the PKD kidneys (Figure 7A–G), indicating the down-regulation of mTOR and MAPK signaling pathways. Moreover, the expression of PCNA (Figure 7H,I) and Ki-67 (Figure 7J,K) was also down-regulated by obacunone, suggesting the retardation of abnormal cell proliferation in the PKD kidneys.

## 4. Discussion

The purpose of this study was to discover therapeutic drugs with strong efficacy and low side effects for ADPKD based on the importance of antioxidant signaling pathways in ADPKD progression. Obacunone was identified from a natural antioxidant compound library, which showed strong inhibitory effect on cyst development both *in vivo* and *in vitro*.

Obacunone is a triterpenoid limonoid compound, which is primarily found in the *Rutaceae* family plants, such as *Phellodendron chinense* and *Tetradium ruticarpum* [18,19]. Previous studies have reported that obacunone exhibits various biological properties, such as anti-inflammation [18], anti-tumor [33] and anti-virus [18,34]. Besides, the antioxidant capacity of it is most recognized. Obacunone acts as a NRF2 agonist by inhibiting its ubiquitination and promoting its nuclear translocation. It is reported that obacunone shows ROS scavenging ability in numerous oxidative stress-related diseases by activating NRF2 [17,19,35,36].

ADPKD is one of the oxidative stress-related diseases [13]. Oxidative stress elevates in renal tissues of ADPKD models as well as human patients [15,24,37,38,39,40]. Accumulation of reactive oxygen species is regarded as an important second hit factor that causes *Pkd* gene mutations and promotes cystogenesis [15,41]. Antioxidant defenses dysfunction is the main cause of oxidative stress in ADPKD. NRF2, as an important antioxidant protein, was found to be inactivated in ADPKD mouse models [24]. NRF2 knockout significantly accelerated renal cyst development [24]. In contrast, pharmacological activation of NRF2 effectively inhibited the progression of ADPKD [24]. Consistent with these investigations, we found that obacunone treatment for 3 days significantly promoted the nuclear translocation of NRF2 in PKD mice, which suppressed oxidative stress in ADPKD.

However, we did not find an activation effect of obacunone treatment for 5 days on NRF2 in PKD mice. In PKD mice, KEAP1 and GSK-3β are up-regulated, which promotes the degradation of NRF2 by activating KEAP1-CUL3 and β-TrCP-CUL1 pathways [24]. Obacunone treatment sharply promoted the nuclear translocation of NRF2 in the early stage of ADPKD, which resulted in the exhaustion of NRF2 at the same time. These processes prevented obacunone from activating NRF2 in PKD mice at postnatal day 5. Indeed, we did find down-regulation of total NRF2 expression in the obacunone-treated PKD mice at postnatal day 5 (data not shown).

NRF2 functions as a transcription factor that is responsible for the expression of antioxidants proteins and phase II detoxification enzymes [42], such as GPX4, HO-1 and NQO-1 [43]. These proteins show potent ROS scavenging abilities, and generally dysfunction in oxidative stress process [42]. In our study, we found that GPX4 was down-regulated as the decline of NRF2 activity in PKD kidneys, implying that GPX4 is involved in the progress of ADPKD. GPX4 is a key enzyme in regulating glutathione metabolism. Its dysfunction leads to glutathione metabolism disorder and directly promotes lipid peroxidation [43,44,45]. Lipid peroxidation elevation was found to promote cyst expansion in ADPKD [14,29]. In our study, it was found that obacunone reversed the down-regulation of GPX4, which restored normal glutathione metabolism and suppressed lipid peroxidation.

Lipid peroxidation mainly results from oxidative stress. Accumulated ROS attacks polyunsaturated fatty acid (PUFA)-containing phospholipids, producing large amounts of lipid peroxides (LPOs) that have adverse effects on cellular homeostasis [46]. MDA and 4-HNE are two most widely studied LPOs, which increase sharply in the kidney of ADPKD mouse model [29]. MDA and 4-HNE have been confirmed as the activators of mTOR and MAPK signaling pathways [28,30,31,32,46]. These two signaling pathways play critical roles in promoting ADPKD progression [1]. Activation of MAPK and mTOR was found in various experimental models as well as human patients [1,47]. Our results showed that obacunone reduced the MDA and 4-HNE contents in the PKD kidneys, followed by down-regulation of mTOR and MAPK signaling pathways. These processes significantly inhibited abnormal proliferation of renal cells.

**Figure 8:**
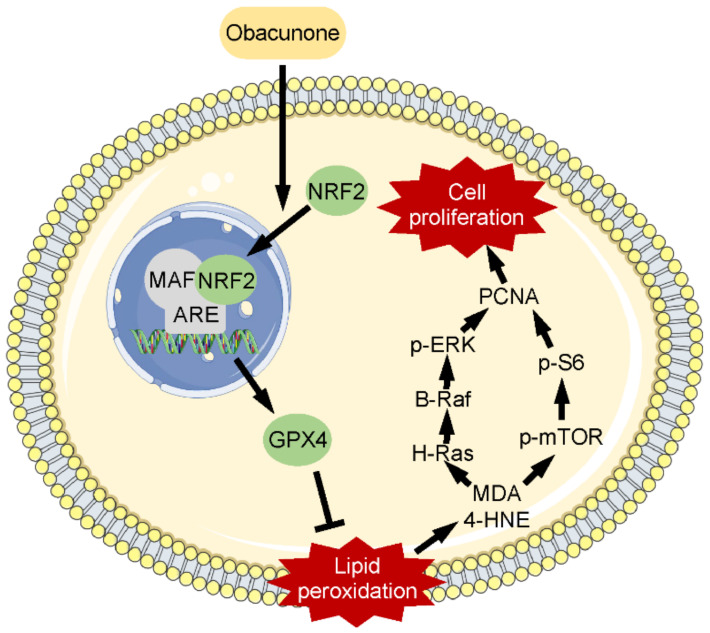
The suggested pharmacological mechanism of obacunone in ADPKD. See text for details.

Our study revealed an important pharmacological mechanism that obacunone regulates the NRF2/GPX4/LPOs pathway in ADPKD. The possible mechanism is diagramed in Figure 8, which suggests that obacunone promotes the nuclear translocation of NRF2, which facilitates the expression of GPX4. The up-regulation of GPX4 suppresses lipid peroxidation accompanied by a decline of 4-HNE and MDA, which down-regulates mTOR and MAPK signaling pathways. These processes finally retard abnormal cell proliferation and cyst expansion.

However, some limitations of this study should be acknowledged. First, due to the limited experimental conditions, we did not generate a *Pkd1* and *Nrf2* double-knockout mouse model to verify the targeting effect of obacunone on NRF2. Second, we did not investigate the effect of obacunone on ferroptosis, even though lipid peroxidation is considered as the trigger of ferroptosis [44]. Elevated ferroptosis level was found in ADPKD mouse models [29], but how ferroptosis accelerates cyst expansion still needs to be clarified. Therefore, it will be a research interest for further studies.

## 5. Conclusions

Our study revealed the therapeutical effect of obacunone on ADPKD. It is the first study that suggests the application of obacunone on ADPKD treatment. The advantages of low cytotoxicity, easy availability and potent efficacy make obacunone ideal to be developed as a candidate therapeutic drug for ADPKD. The druggability of obacunone should be evaluated in further preclinical tests and clinical trials.

## Data Availability

All data produced in this study is provided in the manuscript.
